# Prefrontal cortex, dorsomedial striatum, and dentate gyrus are necessary in the object-based attention test in mice

**DOI:** 10.1186/s13041-020-00711-4

**Published:** 2020-12-14

**Authors:** Bolati Wulaer, Kazuo Kunisawa, Hisayoshi Kubota, Willy Jaya Suento, Kuniaki Saito, Akihiro Mouri, Toshitaka Nabeshima

**Affiliations:** 1 Advanced Diagnostic System Research Laboratory, Fujita Health University Graduate School of Health Science, Toyoake, Aichi Japan; 2 Department of Disease Control and Prevention, Fujita Health University Graduate School of Health Science, Toyoake, Aichi Japan; 3 Department of Regulatory Science for Evaluation & Development of Pharmaceuticals & Devices, Fujita Health University Graduate School of Health Science, 1-98 Dengakugakubo, Kutsukake-cho, Toyoake, Aichi 470-192 Japan; 4 Department of Psychiatry, Hasanuddin University, Makassar, South Sulawesi Indonesia; 5 Japanese Drug Organization of Appropriate Use and Research, Nagoya, Aichi Japan

**Keywords:** Prefrontal cortex, Striatum, Dentate gyrus, Attention, OBAT, c-Fos, Lesion

## Abstract

Disturbances of attention are a common behavioral feature associated with neuropsychiatric disorders with largely unknown underlying causes. We previously developed an object-based attention test (OBAT) as a simple and practical method for evaluating attention in mice. Since its establishment, the test has become a popular method for assessing attention and related underlying mechanisms in various mouse models. However, the underlying neuronal network involved in this test has yet to be studied. The purpose of this study was to identify the principal brain regions activated in the OBAT. Accordingly, C57BL/6J mice were subjected to the OBAT and thereafter prepared for immunohistochemical quantification of c-Fos, an immediate early gene that is frequently used as a marker of neuronal activity, in 13 different brain regions. The number of c-Fos-positive cells was significantly higher in the prefrontal cortex (PFC), dorsomedial striatum (DMS), and dentate gyrus (DG) in the test group as compared to the control group. The neuronal activation of these brain regions during the OBAT indicates that these brain regions are necessary for the regulation of attention in this test. This was supported by excitotoxic lesioning of these brain regions, leading to impaired attention without causing locomotor dysfunction. This study is one of the first attempts to analyze the brain regions that regulate attention in the OBAT. These findings provide an initial insight into the role of these brain regions and ideas for studying the underlying neural and molecular mechanisms.

## Main text

**Fig. 1:**
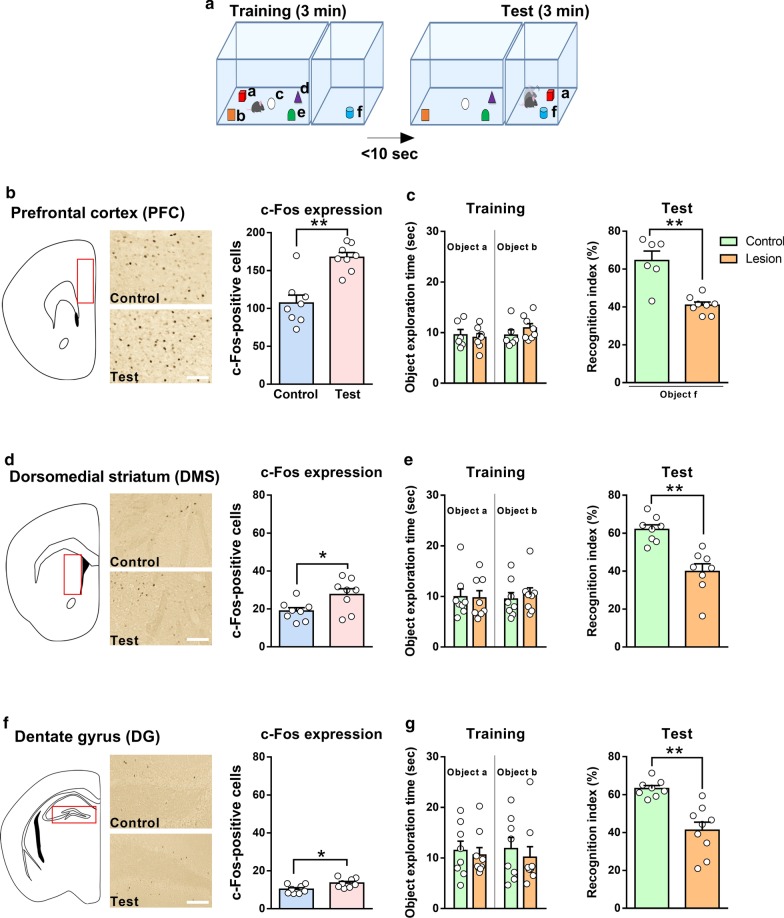
Prefrontal cortex, dorsomedial striatum, and dentate gyrus are necessary for the regulation of attention in the OBAT. **a** OBAT. **b** The representative images and number of c-Fos-positive cells in the PFC of control and PFC lesioned-mice after the OBAT. **c** The performance of control and PFC lesioned-mice in the OBAT. **d** The representative images and number of c-Fos-positive cells in the DMS of control and DMS lesioned-mice after the OBAT. **e** The performance of control and DMS lesioned-mice in the OBAT. **f** The representative images and number of c-Fos-positive cells in the DG of control and DG lesioned-mice after the OBAT. **g** The performance of control and DG lesioned-mice in the OBAT. *p < 0.05, **p < 0.01. n = 6–8 mice each group. Plot data indicate each mouse’s performance. Scale bar indicates 100 μm. The data are expressed as mean ± SEM

Attention plays a critical role in cognition. Impaired attention is often seen in patients with various neuropsychiatric disorders, such as attention-deficit hyperactivity disorder (ADHD), schizophrenia, and major depressive disorder [1–3]. We have previously developed an object-based attention test (OBAT) as a simple and practical method suitable for the evaluation of attention in mice [4]. This behavioral test relies on mice’s inherent behavior to explore novelty in the absence of any instrumental training or external reinforcers. The test comprises two phases: training (familiarization) and testing. In the training session, mice are presented with five different shaped but similarly sized objects for familiarization; a novel and familiar object are then presented during testing (Fig. 1a). The principle here is to use the natural curiosity of mice; when a new object is presented, healthy mice recognize the familiar object and spend more time exploring the novel object during testing. Mice with impaired attention spend approximately equal time with the familiar and novel objects. This is similar to paired comparisons used in object-based visual attention tests in human subjects [5].

Since its establishment, the test has become a popular method to assess attention and related underlying mechanisms in different mouse models [6–8]. Among many tasks that have been developed to assess attentional functions in rodents, OBAT is easy to perform and does not require expensive equipment, and therefore may be accessible to investigators on a tight budget. In currently available tasks for assessing attention in mice, the presence of food/liquid reinforcers associated with the choice stimuli might result in an ambiguous interpretation of animal responses and potential bias in choice decisions, making the interpretation of results uncertain [9]. For example, prenatal nicotine exposure in mice is known to alter reward circuits [6]. One of the marked advantages of OBAT over other attention-related tests is the significantly shortened experimental period (e.g., five-choice serial reaction time task, 3–5 months; OBAT, 1 day) [4, 10]. This further emphasizes OBAT as ideal for the evaluation of attention in younger mice, which is a fundamental factor in research related to some disorders, such as ADHD [6]. These characteristics support the use of OBAT as a test for evaluating attention in preclinical studies. However, the underlying neuronal network involved in this test has yet to be studied.

The first aim of the study was to identify the principal brain regions activated by OBAT. C57BL/6J mice were subjected to the OBAT and sacrificed for immunostaining quantification of c-Fos, an immediate early gene that is frequently used as a marker of neuronal activity, in 13 different brain regions (Additional file 1: Table 1). The number of c-Fos-positive cells was significantly higher in the anterior cingulate cortex (ACC), prelimbic (PrL) and infralimbic (IL) cortices of the prefrontal cortex (PFC; Fig. 1b), dorsomedial striatum (DMS; Fig. 1d), and dentate gyrus (DG; Fig. 1f) in the test group compared to those in the control group (no objects in the test session). To further evaluate the importance of these brain regions, we bilaterally lesioned these regions using excitotoxic ibotenic acid in different batches of mice that underwent the OBAT. Ibotenic acid produces excessive Ca^2+^ through activation of glutamate receptors resulting neuronal cell death, further leads to behavioral changes [11]. There were approximately 50% decreases of neuronal cells in the targeted brain regions (Additional file 2: Figure 1). Mice were given 1 week to fully recover from the surgery. Given the role of the PFC, DMS, and DG in controlling motor function, open field and rota-rod tests were conducted to provide an additional control measure of locomotor function in the lesioned mice (Additional file 2: Figure 1a and b). We confirmed that the impaired attention in lesioned mice was not an artifact of locomotor dysfunction, with no difference between control and lesioned mice in the total distance traveled in the open field or motor skill learning in the rota-rod tests (Additional file 2: Figure 1c, d, f, g, i, j). Mice were subjected to the OBAT on the following day (Fig. 1a). In the training session, control and lesioned mice spent a similar amount of time exploring the objects. In the test session, lesioned mice showed impaired attention, as evidenced by the significantly decreased time spent exploring the novel object (Fig. 1c, e, and g). Statistical analyses were given in the Additional file 1: Table 2.

Taken together, the findings of this study suggest that the PFC, DMS, and DG (but may not be limited to) are necessary for the regulation of attention in the OBAT in mice. These results are consistent with previous studies that the PFC, DMS, and DG are involved in other attention-related tests [12–14]. We have successfully identified brain regions activated during the OBAT using c-Fos mapping and confirmed that these areas are involved in the regulation of attention in a lesion study. It should be kept in mind that sensory ability and motivation processes could also contribute the behavioral performance in this test when interpreting the obtained results. However, we cannot distinguish whether the c-Fos was triggered during training or testing because the interval between the sessions was only 10 s (e.g., information processing). Therefore, it is important to develop improved tools to study the specific cell types in specific neuronal projections during the test (including subregional differences in the PFC) by using more temporally precise manipulations, such as optogenetic manipulations, as well as in vivo recording [15].

Notwithstanding these limitations, this study is one of the first attempts to analyze brain regions activated in the OBAT. These findings provide an initial insight into the roles of these brain regions and suggest how to study the brain circuit interactions and related molecules that contribute to attentional function in normal and disrupted conditions, consequently, suggesting potential treatments for neuropsychiatric disorders.

## Supplementary information

**Additional file 1: Table 1.** Expression of c-Fos in 13 different brain regions in C57/B6 mice. **Table 2.** Statistical analyses used in the manuscript.

**Additional file 2: Figure 1.** Normal locomotor function and decreased percentage of neuronal death in the lesioned mice.

## Data Availability

All data used in this study are available from the corresponding author upon reasonable request.

## Abbreviations

ACC: Anterior cingulate cortex
ADHD: Attention-deficit hyperactivity disorder
DG: Dentate gyrus
DLS: Dorsolateral striatum
DMS: Dorsomedial striatum
IL: Infralimbic
NAc Core: Nucleus accumbens core
NAc Shell: Nucleus accumbens shell
OBAT: Object-based attention test
PFC: Prefrontal cortex
PrL: Prelimbic
