# Supplementary material for: Prefrontal cortex, dorsomedial striatum, and dentate gyrus are necessary in the object-based attention test in mice
Source: Mol Brain. 2020 Dec 14;13:171. doi: 10.1186/s13041-020-00711-4 (PMC7737308; doi:10.1186/s13041-020-00711-4)
Supplement: Supplementary file 1 — Additional file 1: Table 1. Expression of c-Fos in 13 different brain regions in C57/B6 mice. Table 2. Statistical analyses used in the manuscript. [file 13041_2020_711_MOESM1_ESM.docx]

**Additional information**

**Prefrontal cortex, dorsomedial striatum, and dentate gyrus are necessary in the object-based attention test in mice**

Bolati Wulaer, Kazuo Kunisawa, Hisayoshi Kubota, Willy Jaya Suento, Kuniaki Saito, Akihiro Mouri, Toshitaka Nabeshima.

**Materials and Methods**

**Animals**

C57BL/6J mice were purchased from Japan SLC Inc. (Shizuoka, Japan) (RRID: IMSR_JAX:000664). All behavioral experiments were performed using 8-week-old mice during the facility light cycle. Male mice were used in order to avoid potential estrus cycle-related performance variability in female mice (1). The sample size for each experiment was determined based on our previous studies using the relevant type of experiment (2-4). Animals were housed in the animal facilities at the Fujita Health University Graduate School of Medicine under specific pathogen-free conditions, maintained at 25°C on a 12-h light/dark cycle (lights on at 08:00), with free access to food and water. Animal care and use were performed in accordance with the National Institute of Health Guide for the Care and Use of Laboratory Animals and were approved by the Animal Experimentation Committee of Fujita Health University Graduate School of Medicine (Permit Number: APU19119).

**Open field test**

To evaluate the locomotion of mice, an open field test was performed as described in our previous report (4) (Figure 1a) with minor modifications. The open field consisted of a square area with gray walls (42 × 42 × 40 cm) set in a dark, sound-attenuated room. The floor of the field was divided into nine identical squares with a light (200 lux) positioned 100 cm above the center of the floor. Each mouse was placed in one corner of the open field. The mice were allowed to explore the environment freely for 5 min. The total distance traveled was measured using an ANY-maze video tracking system (Cat# 6000, Stoelting Co., Ltd., Wood Dale, IL, USA).

**Rota-rod test**

The motor functions of the mice were examined using the rota-rod test (Cat# 47650, Muromachi Kikai Co., Ltd., Tokyo, Japan; Figure 1b) according to the method outlined in our previous report (4). The test was performed by placing a mouse on a rotating treadmill drum (3 cm diameter) with constant illumination of 20 lux; how long the mouse was able to maintain its balance on the treadmill was measured. Four trials were conducted in which the treadmill rotated at 12 rpm for a maximum of 120 seconds. The mean latency to fall of each mouse was calculated and used in subsequent analyses. The investigator was blinded to the experimental conditions.

**Object-based attention test (OBAT)**

We previously established the OBAT (5). We used a rectangular, two-chambered, opaque plexiglass box including an exploring chamber (40 cm × 40 cm × 22 cm) and test chamber (40 cm × 20 cm × 22 cm). The dividing walls were made from opaque plexiglass with sliding openings that allowed access between each chamber. Briefly, mice were allowed to explore both training and testing chambers for a total of 10 min during the habituation period. In the training session, mice were exposed to five similar-sized but different shaped objects (e.g., objects a-e in Figure 1a) for 3 min. Previous studies have observed that a mouse spends an equal amount of time exploring the five objects during a 3-min exposure (5). Therefore, the time spent exploring two randomly selected objects out of the five was recorded. Mice were immediately (less than 10 seconds) moved to the test session where a familiar and novel object (e.g., object f) were introduced. The recognition index was expressed as the ratio (Tf × 100)/(Ta + Tf), where Ta and Tf are the time spent in the testing session exploring object a and object f, respectively (Figure 1c). The investigator was blinded to the experimental conditions.

**3, 3’-diaminobenzidine (DAB) staining**

DAB staining was performed as described previously (3). Two hours after the start of the OBAT, brains were post-fixed overnight in 4% paraformaldehyde and cryoprotected in 30% sucrose in phosphate-buffered saline (PBS). The brain tissues were prepared (30 μm) using a cryostat (RRID:SCR_016844; Leica CM3050 S Research Cryostat, Germany). The coronal sections were prepared, washed with PBS containing 0.3% Triton X-100, incubated at room temperature (~20-25 °C) for 2 h in the presence of 2% normal goat serum (RRID: AB_2336820; Vector Laboratories Inc., USA), and then incubated with a rabbit anti-c-Fos antibody (RRID: AB_2231974; Synaptic Systems; Germany; 1:1000) at 4°C overnight. After washing with PBS, sections were incubated with a biotinylated goat anti-rabbit IgG secondary antibody (RRID: AB_2336820; Vector Laboratories Inc., diluted 1:1,000) at room temperature for 2 h. The sections were then incubated with PBS containing 0.3% hydrogen peroxide (#08104215; Wako Pure Chemical Industries, Japan) for 30 min in order to inactivate endogenous peroxidase. Thereafter, sections were washed with PBS and incubated with the avidin-conjugated horseradish peroxidase complex (RRID: AB_2336820; Vectastain ABC kit, Vector Laboratories Inc.) at room temperature for 2 h. The signal was visualized using the diaminobenzidine–nickel staining method (3). The sections were stained using 1 mg/mL DAB (#D5637; Sigma-Aldrich), 30% hydrogen peroxide, and 4% nickel chloride (#149-05343; Wako Pure Chemical Industries). c-Fos expression was quantified by an experimenter blinded to the experimental conditions who counted the number of c-Fos-positive cells in 13 different brain regions. The selection of these brain regions was based on a previous paper that focus on forebrain regions to be involved in the cognition with slight changes (6). Control mice were subjected to the OBAT without objects in the test session, otherwise the procedures were similar to the test mice. Only cells that had significant, above background levels of DAB staining in their nuclei were counted (diameter, 7.5–14.5 μm). The acquisition parameters were kept the same for all images. The number of c-Fos-positive cells was counted within an area of 340 × 260 µm using ImageJ software (RRID:SCR_003070; National Institute of Mental Health, USA). Brain areas were determined according to the mouse brain atlas (7). The average of three slices in each mouse was calculated and used for statistical analysis.

**Bilateral excitotoxic lesion**

Mice were anesthetized with a mixture of anesthetic, muscle relaxant, analgesic, and sedative such as medetomidine (0.3 mg/kg; Domitor®, Nippon Zenyaku Kogyo, Tokyo, Japan), butorphanol (5.0 mg/kg; Vetorphale®, Meiji Seika Pharma, Tokyo, Japan), and midazolam (4.0 mg/kg; Midazolam Sandoz®, Sandoz, Tokyo, Japan), and reversed using atipamezole (0.15 mg/kg; Antisedan®, Nippon Zenyaku Kogyo). Once reflex responses had disappeared, mice were head-fixed in a stereotaxic frame (#CA 91042; David Kopf; USA). Ibotenic acid (# I2765, Sigma) was dissolved in PBS (pH 7.2) at a concentration of 10 μg/1 μL, and then bilaterally injected into the PFC (+1.5 mm AP, ±0.5mm ML and −2.5 mm DV), DMS (+1.3 mm AP, ±1.0 mm ML and −3.0 mm DV), or DG (-1.8 mm AP, ±1.0 mm ML, and −2.9 mm DV) at a volume of 0.3 μL/site at a rate of 0.1 μL/min using three different batches of mice (8). After the injection, the needle was left in place for 5 min to prevent backflow. The control group received the same volume of PBS. Mice were given at least 1 week to recover from the surgery and subsequently subjected to behavioral tests.

**Nissl staining**

Nissl staining was performed to examine the spatial specify of lesions. After all behavioral tests were completed, mice were deeply anesthetized with isoflurane (Cat# 099-06571; Wako, Japan). Once reflex responses had disappeared, mice were transcardially perfused with 4% paraformaldehyde in PBS. Brains were post-fixed overnight in 4% paraformaldehyde and cryoprotected in 30% sucrose in PBS. Sections were cut at 20 µm intervals, and staining was done according to the standard procedure (2). Only neurons with a visible nucleus and the entire outline of the cell were counted in the region of interest. Images were acquired with a light microscope (BZ9000; KEYENCE) and analyzed using ImageJ software (RRID:SCR_003070; National Institute of Mental Health, Bethesda, MD, USA).

**Data analyses**

Statistical analyses were performed using GraphPad Prism 6.0 (GraphPad Software, Inc., CA, USA). All data are expressed as the mean ± SEM. Sample sizes were based on past studies that are cited in the relevant sections of the manuscript and methods. For each set of data to be compared, we first performed normality tests. Differences between two groups were analyzed using a two-tailed Student's *t*-test or Mann-Whitney U-test (parametric or nonparametric data, respectively). Two-way ANOVA tests followed by Tukey’s post hoc test were used for statistical analyses with more than three groups. All mice were randomly divided into experimental groups. Cell-counting and behavioral analyses were performed by a blinded investigator. No data points were excluded from any of the experiments unless mice were died during or after the stereotaxic procedure. The criterion for a significant difference was **p* < 0.05 and ***p* < 0.01 in all statistical evaluations.

**Additional Table 1: Expression of c-Fos in 13 different brain regions in C57/B6 mice.**

| **Brain regions** | **Control**  **(n=8)** | **Test**  **(n=8)** | **Student’s**  ***t*-test** | **Mouse brain coordinates based on (7)** | |
| --- | --- | --- | --- | --- | --- |
| Anterior cingulate cortex (ACC) | 23.6±5.05 | 39.6±4.64 | * | AP=+1.54, ML=±0.50, DV=−2.00 | |
| Prelimbic cortex (PrL) | 43.4±2.78 | 63.2±4.46 | ** | AP=+1.54, ML=±0.50, DV=−2.50 | |
| Infralimbic cortex (IL) | 40.1±4.19 | 64.7±3.54 | ** | AP=+1.54, ML=±0.50, DV=−3.00 | |
| Dorsomedial striatum (DMS) | 18.9±1.81 | 27.6±3.06 | * | AP=+1.10, ML=±0.80, DV=−3.00 | |
| Dorsolateral striatum (DLS) | 4.1±0.93 | 4.3±0.66 | n/s | AP=+1.10, ML=±2.10, DV=−3.00 | |
| Nucleus accumbens core (NAc Core) | 11.0±3.28 | 5.7±1.20 | n/s | AP=+1.54, ML=±0.75, DV=−4.50 | |
| Nucleus accumbens shell (NAc Shell) | 6.2±2.31 | 4.1±1.25 | n/s | AP=+1.54, ML=±0.50, DV=−4.80 | |
| CA1 | 0.3±0.16 | 1.1±0.51 | n/s | AP=−1.46, ML=±1.00, DV=−1.50 | |
| CA3 | 3.6±0.59 | 4.0±0.64 | n/s | AP=−1.80, ML=±2.10, DV=−2.20 | |
| Dentate gyrus (DG) | 10.3±0.80 | 13.6±0.87 | * | AP=−1.80, ML=±1.00, DV=−2.00 | |
| Thalamus | 6.3±0.80 | 8.8±1.69 | n/s | AP=−1.46, ML=±0.20, DV=−3.50 | |
| Hypothalamus | 2.1±0.44 | 3.1±0.43 | n/s | AP=−1.46, ML=±0.20, DV=−5.50 | |
| Amygdala | 17.6±4.07 | 16.8±4.10 | n/s | AP=−1.46, ML=±0.28, DV=−4.95 | |
| The number of c-Fos-positive cells were significantly higher in the ACC, Prl, IL, DMS, and DG regions in the brain of test mice. n = 8 mice. The data are expressed as mean ± SEM of c-Fos-positive cells. Significance was calculated for control versus test groups using Student’s t test. **p* <0.05; ***p* <0.01. n/s indicates not significant. | | | | |  |

**Additional Table 2: Statistical analyses used in the manuscript.**

| **Figure** | **Statistical test** | ***p*** **value** | **Significance** |
| --- | --- | --- | --- |
| 1b | Student’s *t*-test | t(14) = 4.90, *p < 0.01* | ** |
| 1c | Two-way ANOVA/ Tukey's post-hoc test | Groups, F (1, 12) = 0.17, *p* = 0.70; objects, F (1, 12) = 2.40, *p* = 0.15; groups × objects interaction, F (1, 12) = 2.70, *p* = 0.13 | n/s |
| 1c | Mann–Whitney U test | *p* < 0.01 | ** |
| 1d | Student’s *t*-test | t(14) = 2.46, *p* < 0.05 | * |
| 1e | Two-way ANOVA/ Tukey's post-hoc test | Groups, F (1, 28) = 0.05, *p* = 0.85; objects, F (1, 28) = 0.01, *p* = 0.15; groups × objects interaction, F (1, 28) = 0.15, *p* = 0.70 | n/s |
| 1e | Mann–Whitney U test | *p* < 0.01 | ** |
| 1f | Student’s *t*-test | t(14) = 2.80, *p* < 0.05 | * |
| 1g | Two-way ANOVA/ Tukey's post-hoc test | Groups, F (1, 30) = 0.46, *p* = 0.69; objects, F (1, 30) = 0.71, *p* = 0.15; groups × objects interaction, F (1, 30) = 0.04, *p* = 0.83 | n/s |
| 1g | Mann–Whitney U test | *p* < 0.01 | ** |
| S1c | Mann–Whitney U test | *p = 0.92* | n/s |
| S1d | Two-way ANOVA/ Tukey's post-hoc test | Groups, F (1, 12) = 0.07, *p* = 0.80; trials, F (3, 36) = 7.39, *p* = 0.01; groups × trials interaction, F (3, 36) = 0.90, *p* = 0.45 | n/s |
| S1e | Mann–Whitney U test | *p* < 0.01 | ** |
| S1f | Mann–Whitney U test | *p* = 0.93 | n/s |
| S1g | Two-way ANOVA/ Tukey's post-hoc test | Groups, F (1, 7) = 0.33, *p* = 0.58; trials, F (3, 21) = 50.63, *p* < 0.01; groups × trials interaction, F (3, 21) = 0.30, *p* = 0.82 | n/s |
| S1h | Mann–Whitney U test | *p* < 0.01 | ** |
| S1i | Mann–Whitney U test | *p* = 0.53 | *n/s* |
| S1j | Two-way ANOVA/ Tukey's post-hoc test | Groups, F (1, 60) = 0.31, *p* = 0.58; trials, F (3, 60) = 15.75, *p* < 0.0001; groups × trials interaction, F (3, 60) = 0.32, *p* = 0.80 | n/s |
| S1k | Mann–Whitney U test | *p* < 0.01 | ** |

**Additional Figure 1. Normal locomotor function and decreased percentage of neuronal death in the lesioned mice**

(a) Open-field test. (b) Rota-rod test. (c) The total distance traveled and (d) motor skill learning ability of control and PFC-lesioned mice. (e) The representative images and percentage of neuronal death in the PFC of control and PFC-lesioned mice. (f) The total distance traveled and (g) motor skill learning ability of control and DMS-lesioned mice. (h) The representative images and percentage of neuronal death in the PFC of control and DMS-lesioned mice. (i) The total distance traveled and (j) motor skill learning ability of control and DG-lesioned mice. (k) The representative images and percentage of neuronal death in the PFC of control and DG-lesioned mice. ***p* < 0.01. n = 6–8 mice each group. Plot data indicate each mouse’s performance. Scale bar indicates 100 μm. The data are expressed as mean ± SEM.

**References**

1. Meziane H, Ouagazzal AM, Aubert L, Wietrzych M, Krezel W. Estrous cycle effects on behavior of C57BL/6J and BALB/cByJ female mice: implications for phenotyping strategies. Genes Brain Behav. 2007;6(2):192-200.

2. Wulaer B, Kunisawa K, Hada K, Suento WJ, Kubota H, Iida T, et al. Shati/Nat8l deficiency disrupts adult neurogenesis and causes attentional impairment through dopaminergic neuronal dysfunction in the dentate gyrus. J Neurochem. 2020;10.1111/jnc.15022.

3. Wulaer B, Nagai T, Sobue A, Itoh N, Kuroda K, Kaibuchi K, et al. Repetitive and compulsive-like behaviors lead to cognitive dysfunction in Disc1(Delta2-3/Delta2-3) mice. Genes Brain Behav. 2018;17(8):e12478.

4. Suento WJ, Kunisawa K, Wulaer B, Kosuge A, Iida T, Fujigaki S, et al. Prefrontal cortex miR-874-3p prevents lipopolysaccharide-induced depression-like behavior through inhibition of indoleamine 2,3-dioxygenase 1 expression in mice. J Neurochem. 2020.

5. Alkam T, Hiramatsu M, Mamiya T, Aoyama Y, Nitta A, Yamada K, et al. Evaluation of object-based attention in mice. Behav Brain Res. 2011;220(1):185-93.

6. Brigman JL, Daut RA, Wright T, Gunduz-Cinar O, Graybeal C, Davis MI, et al. GluN2B in corticostriatal circuits governs choice learning and choice shifting. Nat Neurosci. 2013;16(8):1101-10.

7. Paxinos G, Franklin KBJ. The mouse brain in stereotaxic coordinates: Elsevier Academic Press; 2004.

8. Kim S, Matyas F, Lee S, Acsady L, Shin HS. Lateralization of observational fear learning at the cortical but not thalamic level in mice. Proc Natl Acad Sci U S A. 2012;109(38):15497-501.
