# Supplementary figures and images for: Prefrontal cortex, dorsomedial striatum, and dentate gyrus are necessary in the object-based attention test in mice
Source: Mol Brain. 2020 Dec 14;13:171. doi: 10.1186/s13041-020-00711-4 (PMC7737308; doi:10.1186/s13041-020-00711-4)

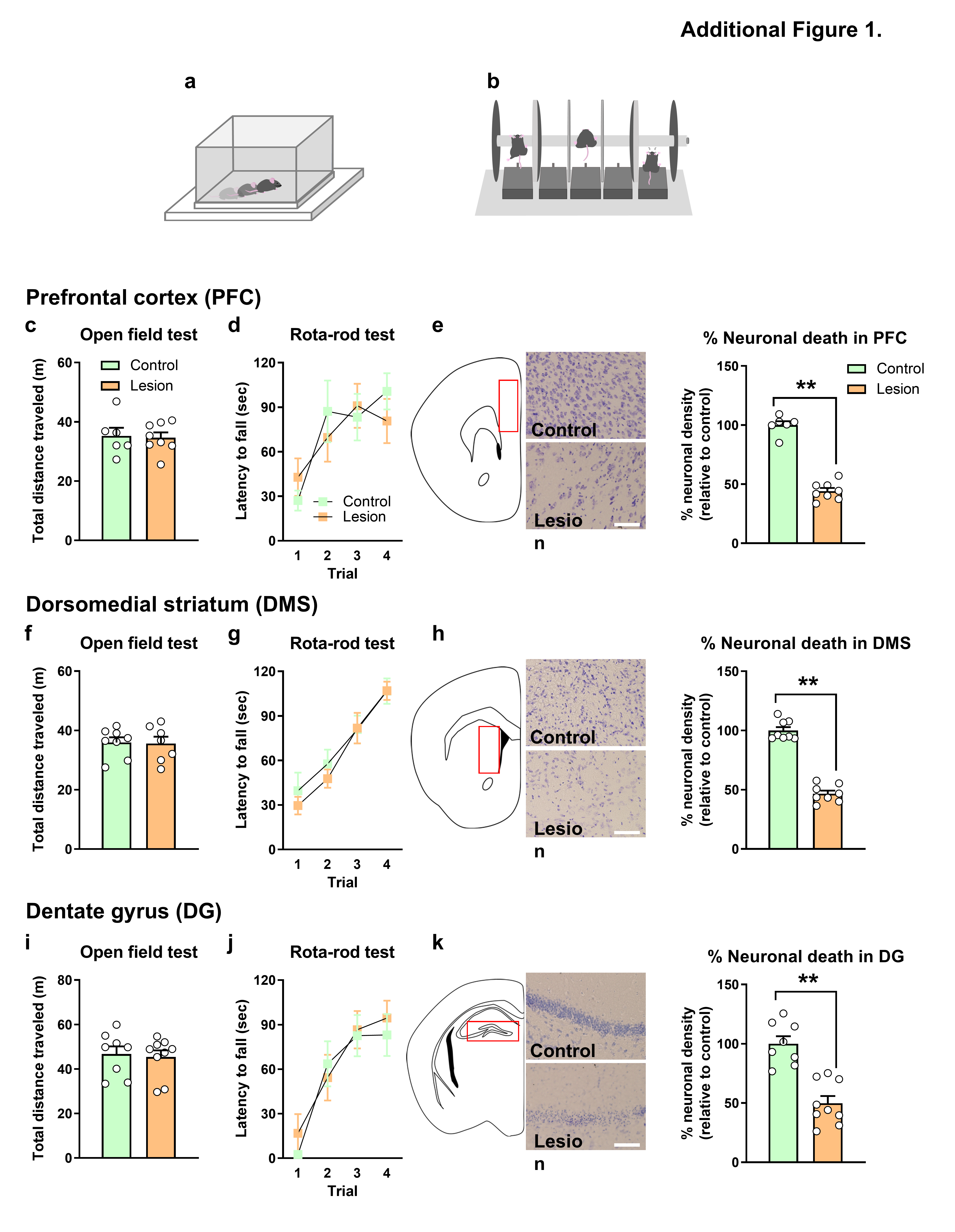

Supplement: Supplementary file 2 — Additional file 2: Figure 1. Normal locomotor function and decreased percentage of neuronal death in the lesioned mice. [file 13041_2020_711_MOESM2_ESM.tif]
